# Production and processing of antioxidant bioactive peptides: A driving force for the functional food market

**DOI:** 10.1016/j.heliyon.2020.e04765

**Published:** 2020-08-28

**Authors:** Solomon Abebaw Tadesse, Shimelis Admassu Emire

**Affiliations:** a Department of Food Science and Applied Nutrition, College of Applied Sciences, Addis Ababa Science and Technology University, P.O.Box 16417, Addis Ababa, Ethiopia; b Department of Food Engineering, School of Chemical and Bioengineering, Addis Ababa Institute of Technology, Addis Ababa University, Ethiopia

**Keywords:** Food science, Food technology, Antioxidant peptides, Functional foods, Enzyme hydrolysis, Fermentation, Emerging processing technologies, Bioinformatics

## Abstract

Recently, the demand for functional foods in the global market has increased rapidly due to the increasing occurrences of non-communicable diseases and technological advancement. Antioxidant peptides have been suggested as ingredients used to produce health-promoting foods. These peptides are encrypted from various food derived protein sources by chemical and enzymatic hydrolysis, and microbial fermentation. However, the industrial-scale production of antioxidant peptides is hampered by different problems such as high production cost, and low yield and bioactivity. Accordingly, novel processing technologies, such as high pressure, microwave and pulsed electric field, have been recently emerged to overcome the problems associated with the conventional hydrolysis methods. This particular review, therefore, discussed the current processing technologies used to produce antioxidant peptides. The review also suggested further perspectives that should be addressed in the future.

## 1 Introduction

The emphasis about foods has shifted from providing the essential nutrients for sustaining life and growth to preventing or indeed curing various forms of diseases. Moreover, the recent technological advancement, population lifestyle changes and socio-economic trends throughout the world indicate the need for foods with increased health benefits (Betoret et al., 2011). These are the key determining and driving forces for the growth of the current development and production of functional foods in the global market.

The health benefit of functional foods is derived from the bioactive compounds, such as phytochemicals, vitamins and peptides, found naturally in them, formed during processing, or extracted from other sources and added to them (Butnariu and Sarac, 2019). Among these bioactive compounds, antioxidant peptides have received significant attention in the food industry in recent times. Antioxidant peptides are specific protein fragments possessing antioxidant activity, thus, can be utilized to maintain human health, and food safety and quality by mitigating oxidative stress and lipid peroxidation caused by free radicals generated during oxidation reactions of a human body and food products (Antolovich et al., 2002; Bashir et al., 2017; Gil-chavez et al., 2013; Hema et al., 2017). The main mechanisms by which antioxidant peptides inhibit oxidation are through inactivation of reactive oxygen species, scavenging free radicals, chelating prooxidative transition metals and reduction of hydroperoxides (Elias et al., 2008).

Various food derived protein sources have been utilized to produce bioactive peptides. For example, plant sources, such as walnut meal proteins (Feng et al., 2018), hazelnut protein (Liu et al., 2018), sesame protein (Lu et al., 2019), perilla seed protein (Yang et al., 2018), soybean (Chen et al., 2018), common bean (Chen et al., 2019, Chen et al., 2019) and protein from cauliflower by-products (Montone et al., 2018a); animal sources, such as goat, sheep and bovine milk proteins (Moreno-montoro et al., 2018; Tagliazucchi et al., 2018; Zanutto-elgui et al., 2019), egg (Wang et al., 2018; Eckert et al., 2018) and ham (Xing et al., 2018); fish and their by-products, such as salmon (Neves et al., 2017), stone fish protein (Auwal et al., 2017), chub marckerel (Bashir et al., 2017), turbot skin (Fang et al., 2017), shrimp shell discards (Ambigaipalan and Shahidi, 2017), tilapia frame and skin (Huang et al., 2015); and microalgae proteins, such as blue-green algae (Seddek et al., 2019), Irish brown seaweed *Ascophyllum nodosum* (Kadam et al., 2016), *Tetradesmus obliquus* microalgae (Montone et al., 2018b), have been reported as sources of bioactive peptides.

Conventional and bioinformatic approaches are widely employed to investigate *in vitro* bioactivity and physicochemical properties of antioxidant peptides possibly obtained from selected protein sources (Tejano et al., 2019; Yang et al., 2017). The peptides are inactive since they are fused in their parent proteins via peptide bonds. Accordingly, they must be cleaved from the intact parent protein either chemically or biochemically to get the required bioactivity (Ozyurt et al., 2017; Wang et al., 2017b). In order to produce antioxidant peptides from protein sources, enzymatic hydrolysis and fermentation are preferred over the chemical hydrolysis method due to their GRAS nature (He et al., 2019; Jemil et al., 2016). However, these biochemical methods are not feasible to produce the peptides at the industrial level with higher yield and low cost. In these regards, novel processing technologies, such as high hydrostatic pressure (HHP), microwave processing and pulsed electric field, have recently been emerged as the most promising technologies that can possibly be coupled with biochemical hydrolysis methods to produce antioxidant peptides with better yield and bioactivities in a shorter time and lower cost than biochemical methods (Chian et al., 2019; Dong et al., 2019; Ketnawa et al., 2018; Ma et al., 2018).

In general, the demand for functional foods in the global market has increased due to the fact that the advancement of processing and production technologies with people's awareness about the importance of bioactive peptides as health-promoting ingredients. Therefore, this review presents the current production and processing technologies of antioxidant peptides as driving forces for the development of functional foods.

## 2 Antioxidant peptides

Reactive oxygen species (ROS), such as superoxide anion (•O_2_^−^), hydroxyl radicals (•OH) and hydrogen peroxide (H_2_O_2_), are highly reactive molecules that are formed endogenously (physiologically) or exogenously (non-physiologically). Physiologically, ROS are generated as by-products during the oxidation reaction of an organism's metabolism with the help of different intracellular enzymes, such as NADPH oxidases and lipoxygenases (Ahmad et al., 2017; Dharmaraja, 2017). ROS can also be formed non-physiologically through the action of different environmental agents, such as pollutants, ionizing radiations and ultraviolet light (Dayem et al., 2017).

Endogenous ROS have essential physiological functions such as protection of the cell from infection, regulation of intercellular signaling pathways and facilitation of reproduction (Dharmaraja, 2017; Nita and Grzybowski, 2016; Wagner et al., 2019). However, an excessive amount of ROS causes an oxidative stress condition when the living organisms are unable to develop their own antioxidant defense mechanism (Antolovich et al., 2002). Consequently, a considerable number of people in the world have acquired at least one of the different non-communicable diseases, such as diabetes, atherosclerosis and cancer. In addition, lipid peroxidation (LPO), because of ROS, is one of the main causes of the deterioration of oxidation sensitive components and compounds of food products (Zou et al., 2016, Zou et al., 2016).

In the past decades, artificial antioxidants, such as butylated hydroxytoluene (BHT) and butylated hydroxyanisole (BHA), have been used to reduce LPO in food products (Lee et al., 2009). Moreover, some research studies have reported the potential role of these synthetic chemicals to prevent LPO and oxidative stresses in experimental models by restoring the cellular antioxidant enzyme status (Dassarma et al., 2018; Jayalakshmi and Sharma, 1986; Partridge et al., 1983). These synthetic antioxidants are stable to an extreme range of different environmental conditions and low in cost. However, the use of artificial antioxidants in the food industry is under strict regulation due to the potential health effects they exert negatively (Reddy and Urooj, 2005). Therefore, substituting synthetic chemicals by natural bioactive antioxidants has become a key study area.

Antioxidant peptides are among the major functional ingredients receiving an enormous interest by researchers, consumers and the food industry for their potential applications to develop health-promoting foods and maintain the quality and safety of food products (Chai et al., 2017; Zapadka et al., 2017). The amino acid composition, hydrophobicity and sequence, and molecular weight of the peptides determine the bioactivity of peptides once they are released from the parent protein (Zou et al., 2016, Zou et al., 2016). Peptides containing 2–20 amino acids with a molecular weight less than 3 kDa and the presence of hydrophobic amino acids, such as proline, valine, tryptophan and phenylalanine, showed potent antioxidant activity (Ketnawa et al., 2018; Wang et al., 2014; Yang et al., 2019).

Generally, two methods are employed to determine the antioxidant capacity of peptides. The first one is based on hydrogen atom transfer (HAT) in which the capability of an antioxidant compound to scavenge ROS in the substrate by donating hydrogen atom is measured in a competitive reaction (Sohaib et al., 2017). Total radical trapping antioxidant parameter (TRAP), oxygen radical absorbance capacity (ORAC) and carotene bleaching assay are the most common examples of HAT assay (Ambigaipalan and Shahidi, 2017; Zhang and Mu, 2017). The second methods work based on electron transfer in which an antioxidant is estimated by comparing it with a certain oxidant (Zou et al., 2016, Zou et al., 2016). These methods include ferric ion reducing antioxidant power (FRAP), trolox equivalent antioxidant capacity (TEAC), hydroxyl (OH) radical scavenging activity, DPPH radical-scavenging capacity (DPPH), superoxide anion radical-scavenging (O_2_) activity and superoxide dismutase (SOD)-like activity (Barkia, Al-haj, Hamid, Zakaria and Saari, 2018; Chen et al., 2019, Chen et al., 2019; Iskandar et al., 2015; Yang et al., 2018). Tables 1 and 2 shows some examples of antioxidant activity evaluation methods for selected protein hydrolysates and peptides.

**Table 1:** Sources, evaluation assays, and amino acid sequences of antioxidant peptides.

Fish type	Assay	Amino acid sequence	References
Tuna dark muscle by-product	DPPH radical-scavenging capacity and ferric thiocyanate method	LPTSGAALT and PMATMVT	(Hsu, 2010)
Tuna backbone protein	Lipid peroxidation inhibition assay and direct free radical scavenging activity by using electron spin resonance spectrometer	VKAGFAWTANQQLS	(Je et al., 2007)
Flounder fish (*Paralichthys olivaceus*)	DPPH radical scavenging activity, Hydroxyl radical scavenging activity and Peroxyl radical scavenging activity	VCSV and CAAP	(Ko et al., 2013)
Salmon trimmings	Dipeptidyl peptidase IV (DPP-IV) inhibitory and oxygen radical absorbance capacity (ORAC) activities	GPAV, VC and FF	(Neves et al., 2016)
Salmon gelatin (*Salmo salar,* SG)	Dipeptidyl peptidase IV (DPP-IV) inhibitory, oxygen radical absorbance capacity (ORAC)	GGPAGPAV, GPVA, PP and GF	(Neves et al., 2017)
Mung bean (*Vigna radiata* L. Wilczek)	Calcium-binding assay	LLLGI, AIVIL and HADAD	(Budseekoad et al., 2018)
Finger millet	ABTS, DPPH, metal-chelating, and hydroxyl radical scavenging activity	TSSSLNMAVRGGLTR and STTVGLGISMRSASVR	(Agrawal et al., 2019)
Miiuy croaker (*Miichthys miiuy*) muscle	2,2-Diphenyl-1-picrylhydrazyl radical (DPPH) radicals scavenging activity	YASVV, NFWWP, FWKVV, TWKVV and IRWWW	(He et al., 2019)

A = alanine, R = arginine, N = asparagine, D = aspartic acid, C = cysteine, E = glutamic acid, Q = glutamine, G = glycine, H = histidine, I = isoleucine, L = leucine, K = lysine, M = methionine, F = phenylalanine, P = proline, S = serine, T = threonine, W = tryptophan, Y = tyrosine, V = valine.

**Table 2:** Selected sources of peptides, enzymes used for hydrolysis and properties of the peptides.

Source	Hydrolysis method	Anti-oxidative Properties	Reference
Shrimp shell processing discards	Enzymatic hydrolysis (Trypsin, α-chymotrypsin and Pepsin)	Radical scavenging activities (ABTS, DPPH and hydroxyl), reducing power and ferrous ion (Fe^2+^) chelating ability, inhibition of β-carotene bleaching, cupric ion induced LDL cholesterol peroxidation, and peroxyl and hydroxyl radical induced DNA strand scission	(Ambigaipalan and Shahidi, 2017)
Stone fish protein	Enzymatic hydrolysis (Bromelain)	DPPH scavenging activity and ferrous ion- chelating activity	(Auwal et al., 2017)
Chub mackerel (*Scomber japonicas)*	Enzymatic hydrolysis (Alcalase, Protamex and Neutrase)	DPPH and ABTS radical-scavenging activity	(Bashir et al., 2017)
*Acetes indicus*	Enzymatic hydrolysis (Alcalase)	DPPH radical-scavenging activity and ferrous ion chelating ability	(Dhanabalan et al., 2017)
Tuna dark muscle by-product	Enzymatic hydrolysis (Rientase and Peptidase XXIII)	DPPH radical-scavenging capacity, ferric thiocyanate anti-oxidative activity	(Hsu, 2010)
Sandfish (*Arctoscopus japonicus*) meat and roe	Enzymatic hydrolysis (Alcalase, Flavourzyme, Protamex, Neutrase and Collupulin)	DPPH radical scavenging activity	(Jang et al., 2017)
Male silkmoth	Enzymatic hydrolysis (Alcalase)	DPPH and ORAC radical-scavenging capacity	(Liu et al., 2016)
Anchovies (*Ilisha melastoma*)	Fermentation (spontaneous)	DPPH and ABTS radical scavenging capacity	(Najafian and Babji, 2018)
Sweet potato	High hydrostatic pressure (HHP) assisted Alcalase digestion	ORAC radical-scavenging capacity	(M. Zhang and Mu, 2017)
Cow Milk (Whey Protein)	High hydrostatic pressure (HHP) assisted Trypsin, Pepsin, Chymotrypsin and Peptidase	Ferric reducing antioxidant power (FRAP)	(Iskandar et al., 2015)
Microalgae (*Chlorella Sorokiniana*)	Bioinformatics Analyses	Dipeptidyl peptidase-IV (DPP IV) inhibition	(Tejano et al., 2019)
Microalgal	Enzymatic hydrolysis (Trypsin, Flavourzyme, Papain and Pepsin)	2,2-Diphenyl-1-picrylhydrazyl (DPPH) scavenging activity, Metal chelating activity	(Barkia et al., 2018)
Black soybean [*Glycine max* (L.) Merr.] byproduct	Enzymatic hydrolysis (Alcalase)	2,2-Diphenyl-1-picrylhydrazyl (DPPH) scavenging activity, Hydroxyl radical scavenging activity and Ferric reducing antioxidant power (FRAP)	(Z. Chen et al., 2018)

## 3 Source of antioxidant peptides

Bioactive peptides can be obtained from various food-derived plants, animal and marine protein sources (Tables 1, 2, and 3). In the search for antioxidant peptides, however, protein amount and quality, and cost of the raw materials have an important effect. Therefore, those cheap and simply available materials such as industrial byproducts containing a high amount of protein can feasibly be used to produce antioxidant peptides.

Accordingly, protein hydrolysates and peptides from various cheap and underutilized marine sources, such as fish muscles and byproducts, and microalgae have been widely used to produce antioxidant protein hydrolysates and peptides. For example, Atlantic salmon (Auwal et al., 2017), Chub marckerel (Bashir et al., 2017), *Acetes indicus* (Dhanabalan et al., 2017), *Epinephelus malabaricus* skin (Hema et al., 2017), Turbot skin (Fang et al., 2017), Shrimp shell discards (Ambigaipalan and Shahidi, 2017), Tilapia frame and skin (Huang et al., 2015), Blue-green algae (Seddek et al., 2019), Irish brown seaweed (Kadam et al., 2016), *Tetradesmus obliquus* microalgae (Montone et al., 2018b) have been reported as sources of antioxidant peptides.

A wide range of different plant and animal products peptides, for example, walnut protein (Feng et al., 2018), hazelnut protein (Liu et al., 2018), sesame protein (Lu et al., 2019), rice bran protein (Wattanasiritham et al., 2016), perilla seed protein (Yang et al., 2018), soybean (Chen et al., 2018), common bean (Chen et al., 2019), cauliflower by-products (Montone et al., 2018a), goat milk (Moreno-montoro et al., 2018), sheep milk (Tagliazucchi et al., 2018), bovine milk (Padghan et al., 2017; Zanutto-elgui et al., 2019), egg (Eckert et al., 2018; Wang et al., 2018), ham (Xing et al., 2018) can also be used as sources of antioxidant.

## 4 Production of antioxidant peptides

**Figure 1:**
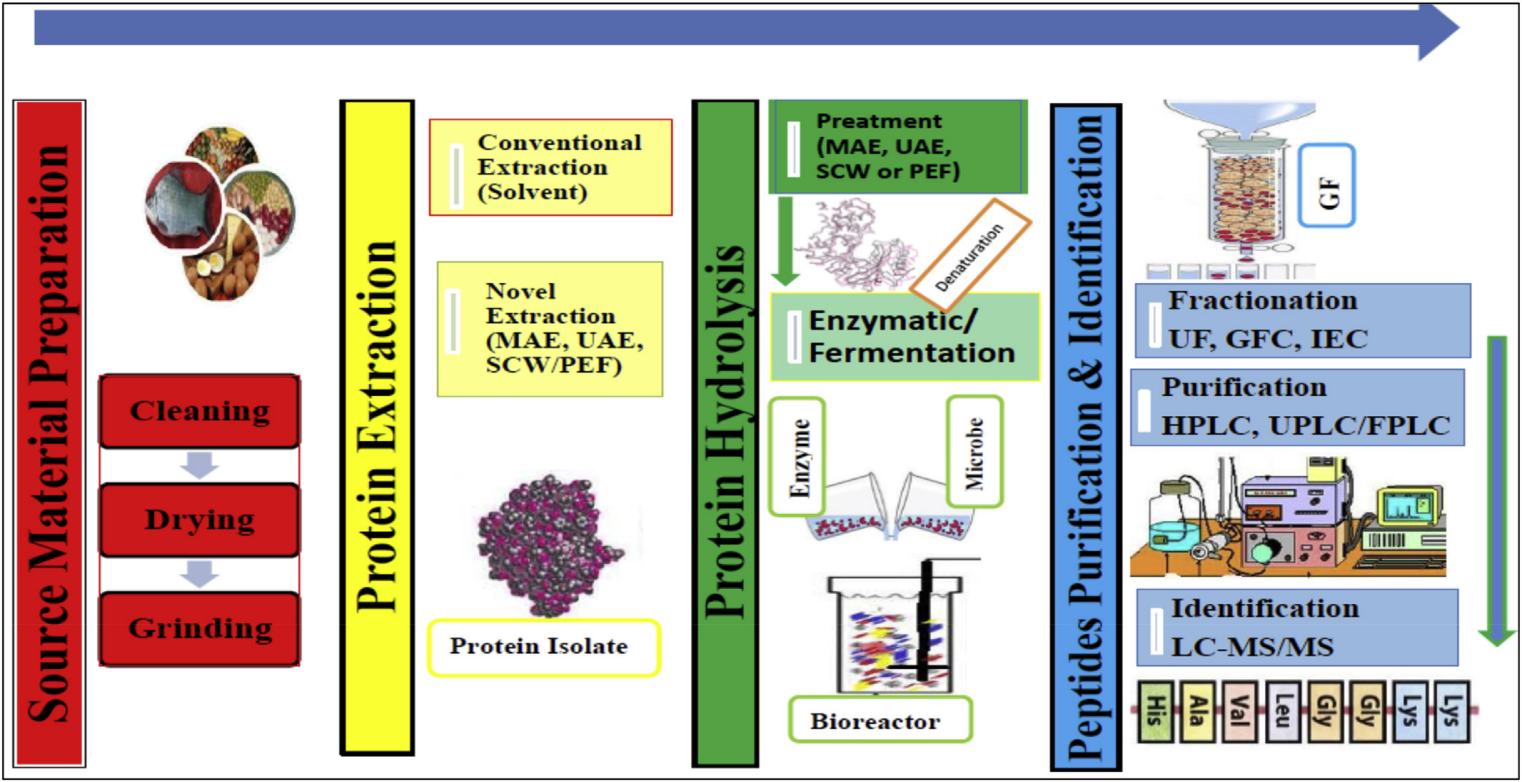
Schematic representation of the production of antioxidant peptide.

The production of antioxidant peptides involves numerous steps including preparation and isolation of protein from the source material, hydrolysis of the isolated or concentrated protein, and purification and identification of peptides from the hydrolyzed protein (Figure 1). The isolation of antioxidant peptides from protein sources is mostly carried out in two major approaches namely: conventional (experimental based) and bioinformatic (*in silico*) (He et al., 2019; Tejano et al., 2019).

### 4.1 Conventional (experimental based) approach

This approach is a common method used to isolate antioxidant peptides. This method is carried out in laboratories where the peptides are cleaved from the parent proteins through experiment trial and error routines (Hsu, 2010). The method involves preliminary processes to concentrate and prepare proteins, protein hydrolysis to release peptides, and purification and identification of peptides with specific bioactivities (Neves et al., 2017; Wang et al., 2014).

#### 4.1.1 Preliminary processes

Biochemical hydrolysis methods require pure protein as much as possible to control the hydrolysis operation and release peptides having antioxidant activities (Admassu et al., 2017). Therefore, components of the raw material other than protein must be separated using a set of different procedures. The choice of preliminary processing method, however, depends on the type, interactions between components, and nature and structure of proteins in the source materials (Wang et al., 2017b).

Protein in plant materials and algae exists in the form of protein bodies with other components, such as carbohydrates, lipids and fibers, under a high and rigid structural complex cell wall (Wang et al., 2016). Conventionally, protein is extracted from the source materials using sufficient quantities of organic and inorganic solvents, which facilitate the removal of lipid and soluble matters, after the materials are cleaned, dried and milled (Chen et al., 2018; Seddek et al., 2019). Then, the defatted material is mixed with deionized water containing alkaline solution, to maintain the pH at approximately 10, and centrifuged to separate the supernatant containing the protein from insoluble solids, such as carbohydrates, fiber and minerals (Yang et al., 2018). The isoelectric precipitation method is used to concentrate the protein after adjusting the pH of the supernatant by adding a sufficient amount of acid or base solution (Chen et al., 2018). Finally, the protein isolate is dried using a suitable dryer, commonly freeze dryer.

Protein extraction from fish and animal products is not that much difficult like obtaining protein isolate from plant and algae sources. The preliminary process involves cleaning of the source materials thoroughly with tap water to remove any foreign maters and separating the meat from the skin and bones (Dhanabalan et al., 2017; Hema et al., 2017). Afterward, the required part of the materials are mixed with distilled water containing sufficient amount of solvent, commonly Hexane, and centrifuged to remove oil components (Bashir et al., 2017).

#### 4.1.2 Hydrolysis of protein concentrates

Although chemical hydrolysis methods are simple and quick operations, it is challenging to find the required yield and properties of antioxidant peptides since these methods lack sensitivity and specificity. Furthermore, chemical methods are prone to amino acid damages because the sample is treated in extreme pH range at high temperature and, in some cases, at high pressure over a given time (Ishak and Sarbon, 2017; Villamil et al., 2017). According to the review by Villamil et al. (2017), amino acids like tryptophan, methionine and cysteine are destroyed because of the temperature and pressure effect of the hydrolysis process. Moreover, asparagine and glutamine are changed into aspartic acid and glutamic acid, respectively. As a result, formations of salts as a side product of the neutralization process are also the main problem of the chemical hydrolysis method since salts affect the bioactivities and functional properties of designated peptides.

Taking the limitation of the chemical methods, enzyme hydrolysis and fermentation have gained a significant consideration to produce quality protein hydrolysates having better functional properties and bioactivities because they use mild conditions and they are more precise in the cleavage of peptide bonds (Najafian and Babji, 2018; Wang et al., 2017a). Furthermore, there are no or limited side reactions that take place and they are found easily in recovery and purification procedures of the peptides (Auwal et al., 2017; Bashir et al., 2017; Godinho et al., 2015).

##### 4.1.2.1 Enzymatic hydrolysis of protein

The enzymatic hydrolysis process is usually performed by adding one or more peptidases in a reactor containing a mixture of deionized water and concentrated protein after adjusting the main controlling parameters such as temperature and pH (Hsu, 2010). Then, the reaction is continued until a constant degree of hydrolysis obtained (Ko et al., 2013). Maintaining the optimum pH range of the mixture during hydrolysis is the main challenge since the pH is rapidly changed because of the cleavage of peptide bonds and the release of free amino acids (Auwal et al., 2017). In order to maintain the optimum pH range of the mixture, buffer or neutralizing solutions are usually added to the mixture (Neves et al., 2017). At the end of the hydrolysis process, the mixture is heated at a temperature beyond the optimal value of enzymes to inactivate them, and the mixtures are centrifuged to separate the supernatants containing peptides and stored bellow -20 °C or dried by freeze dryer (Je et al., 2007).

Enzyme type is one of the main parameters that determine the hydrolysis process and antioxidant capacity of protein hydrolysate and peptides. Single, double or multiple endogenous or exogenous peptidases can be used to release peptides of interest. Preferably, exogenous peptidases are selected due to their shorter hydrolysis time and better control of hydrolysis operation to obtain antioxidant peptides with the required molecular weight and amino acid composition (Ambigaipalan and Shahidi, 2017; Dhanabalan et al., 2017). Food-grade proteinases obtained from microorganisms such as Alcalase and Protamex (Bashir et al., 2017; Dhanabalan et al., 2017), plant materials such as papain (Hema et al., 2017), and animal sources such as pepsin, chymotrypsin and trypsin (Ambigaipalan and Shahidi, 2017; Jang et al., 2017) are widely used to generate antioxidant peptides. Table 3 shows examples of enzymes used for producing antioxidant peptides.

Jang et al. (2017) evaluated the effect of five peptidases, namely Alcalase, Collupulin, Flavourzyme, Neutrase, and Protamex, on the DPPH radical scavenging activity of hydrolysates of Sandfish protein. The highest DPPH activity was found from the protein hydrolysate prepared using Alcalase 2.4 L. Conversely, DPPH activities of the hydrolysates obtained by Neutrase and Protamex were the lowest. The difference in the DPPH radical activities of the hydrolysates prepared from the five peptidases is reported due to the amino acid compositions difference in the hydrolysates and the dissimilarity in the specificity of the enzymes used. On the other hand, Je et al. (2007) reported that the DPPH radical scavenging activity of tuna backbone hydrolysate prepared by papain was higher than the hydrolysates obtained using Alcalase, α-chymotrypsin, Neutrase, pepsin and trypsin. Another study by Ko et al. (2013) demonstrated that among the flounder fish muscle hydrolysates prepared using eight different enzymes, α-chymotrypsin provided the hydrolysate with the highest DPPH activity and peroxyl radicals capacity, and the pepsin hydrolysate exhibited the lowest hydroxyl radical scavenging activity. The differences among the hydrolysates in antioxidant properties are because these enzymes have different peptide bond cleavage patterns.

Hydrolysis conditions such as pH, temperature and time, also play significant roles in the search for antioxidant peptides. According to the study by Auwal et al. (2017), the hydrolysis pH, temperature and time indicated strong linear and quadratic effects on DPPH scavenging activity, but the enzyme/substrate ratio exerted a linear effect on DPPH scavenging activity of Stonefish protein hydrolysates. On the other hand, the hydrolysis pH, temperature and enzyme/substrate ratio had strong linear and quadratic effects on Fe^2+^ chelating activity. Accordingly, the highest DPPH scavenging activity (48.94%) and Fe^2+^ chelating activity (25.12%) of Stonefish protein hydrolysates were obtained at the pH 6.5, 54^∘^C, enzyme/substrate ratio of 1.5%, and 360 min. Wang et al. (2017a) reported that when the pH increased from 5.5 to 7.5, DPPH scavenging activity of Mackerel protein hydrolysate increased slightly because of the isoelectric point difference of proteins in Mackerel, which affects the solubility of proteins.

##### 4.1.2.2 Fermentation

Fermentation is a cost-effective method for generating bioactive peptides and food-grade proteins hydrolysates through the action of microorganisms (Ozyurt et al., 2017). Microbial fermentation has been reported as an ideal method to produce bioactive peptides at the industrial level as it is economically feasible and more eco-friendly than the enzymatic method (Godinho et al., 2015; Jemil et al., 2016). Moreover, fermentation plays a significant role in improving the organoleptic and physicochemical properties of products (Najafian and Babji, 2018).

Production of antioxidant peptides from various protein sources using the fermentation process involves inoculation of microbial strains into the reactor containing homogeneous mixtures of concentrated protein, water and microbial foods, mostly simple sugars, which can be used by the strains for growth and development (Najafian and Salam, 2018). The protein is mostly broken by microbes activity and peptidases secreted by the action of microbes during fermentation (Rajendran et al., 2018). Therefore, the choice of microbial strains is highly important to generate sufficient and effective peptides with high antioxidant activity (Ozyurt et al., 2017). Accordingly, microbes with high proteolytic activity and specificity are required for cleaving the peptide bonds in the parent protein and producing high numbers of bioactive peptides, respectively (Ozyurt et al., 2017).

Lactic acid bacteria are frequently used to produce antioxidant peptides from different protein sources. Several studies reported the production of antioxidant protein hydrolysates and peptides from the protein of different plant, animal and marine sources, and their industrial by-products fermented by *Bacillus subtilis* A26, *Bacillus amyloliquefaciens* An6, *Streptococcus spp., Lactobacillus brevis, Lactobacillus plantarum, Pediococcus acidilactici and Enterococcus gallinarum* (Godinho et al., 2015; Jemil et al., 2016; Najafian and Babji, 2018; Ozyurt et al., 2017; Rajendran et al., 2018)*.* Najafian and Salam (2018) identified two new antioxidant peptides, Ala-Ile-Pro-Pro-His-Pro-Tyr-Pro and Ile-Ala-Glu-Val-Phe-Leu-Ile-Tre-Asp-Pro-Lys, from Loma fish protein hydrolysate fermented by *Lactobacillus plantarum* IFRPD P15.

Alternatively, some specific proteolytic fungi strains can also be used to produce bioactive peptides with better antioxidant activities (Najafian and Babji, 2018). Fang et al. (2017) demonstrated that the protein hydrolysate made by fermentation of turbot skin by *Aspergillus oryzae* exhibited better antioxidant activity (60.3%) with excellent stabilities in gastrointestinal digestion and wide ranges of pH and temperature conditions than hydrolysates made by *Streptococcus thermophiles*.

#### 4.1.3 Separation, purification and identification of antioxidant peptides

##### 4.1.3.1 Separation and purification of antioxidant peptides

Protein hydrolysates demonstrate better antioxidant activity compared to their parent proteins, and this shows that hydrolysis of protein is necessary for releasing potent peptides. However, hydrolyzed proteins exhibit different biological activities and physicochemical properties depending on their amino acid sequences, net charges and molecular weights (Sila and Bougatef, 2016). Due to this fact, obtaining the desired antioxidant activity from protein hydrolysate has still been a challenge in proteomic researches. Therefore, antioxidant peptides must be separated and purified from protein hydrolysate based on their physicochemical properties, such as molecular weight, net charge, hydrophobicity and amino acid sequence, to improve bioactivity.

Table 3 shows the separation, purification and identification of some antioxidant peptides obtained from different sources. Membrane ultrafiltration and size exclusion chromatography, mostly gel filtration, are widely used to separate antioxidant peptides according to their molecular weights (Ambigaipalan and Shahidi, 2017; Hsu, 2010; Sila and Bougatef, 2016; Wang et al., 2014). Fast performance liquid chromatography (FPLC) on the ion-exchange column can also be used to fractionate antioxidant peptides based on their net charges (Chen et al., 2019). Furthermore, reverse-phase HPLC on a hydrophobic column matrix can be used to purify antioxidant peptides based on the hydrophobic properties of the peptides (Ko et al., 2013; Xie et al., 2015).

**Table 3:** Fractionation, purification and identification of antioxidant peptides.

Protein source	Fractionation and purification method	Identification method	Amino acid sequences	Molecular weight (Da)	references
Tuna dark muscle by-product	Sephadex G-25 GFC and Two step RP-HPLC	Q-TOF-ESI/MS	LPTSEAAKY PMNYMVT	978 756	(Hsu, 2010)
oysters (*Crassostrea talienwhanensis*)	Sephadex G-25 GFC RP-HPLC with a Kromasil C18 (ODS) column	nano-ESI-MS/MS.	PVMGN EHGV	518 440	(Wang et al., 2014)
Salmon gelatin (*Salmo salar*, SG)	Two step RP-HPLC	UPLC – MS/MS	GPVA	-	(Neves et al., 2017)
Tuna Backbone	FPLC and RP-HPLC	Q-TOF ESI/MS/MS	VKAGFAWTANQQLS	1519	(Je et al., 2007)
Salmon (*Salmo salar* L.)	RP-HPLC	LC-ESI-MS/MS	FIKK PHL		Borawska et al., 2015
Zebra blenny	Sephadex G-25 GFC, RP-HPLC	ESI-LC–MS/MS	HHPDDFNPSVH	434.529	Jemil et al. (2016)
Red alga (*Gracilariopsis lemaneiformis*)	Ultrafiltration, GFC, RP-HPLC	UPLC-MS/MS on Q Exactive mass spectrometer	ELWKTF	822	Zhang et al., 2019a, Zhang et al., 2019b
Microalgae (*Isochrysis* *Zhanjiangensis*)	FPLC, RP-HPLC	Q-TQF-ESI/MS	NDAEYGICGF	1088.16	Chen et al., 2019, Chen et al., 2019
The blue-spotted stingray (*Dasyatis kuhlii*)	Ultrafiltration membranes, Sephadex G-25 GFC STRATA SCX-SPE cartridges	UPLC-Q-TOF-ESI/MS	WAFAPA MYPGLA	661.3224 650.3098	Wang et al., 2018
Goat milk	Ultrafiltration membrane	LC- MS/MS_ESI	LYQEPVLGPVRGPFPI YQEPVLGPVRGPFPIL VQSWMHQPPQPLSPT	1780.90 1780.90 1731.84	Mahdi et al., 2018
Rainbow trout (*Oncorhynchus mykiss*) frame	Size Exclusion Chromatography (SEC), HPLC	MALDI-TOF-MS/MS	NGRLGYSEGVM GNRLGYSWDD	1,182.65	Ketnawa et al. (2018)
Jack Bean	*In silico*	*In silico*	IY HL	294.33 238.30	Harvian and Ningrum, 2019
Blue-spotted stingray	Ultrafiltration membranes, Sephadex G-25 GFC, STRATA SCX-SPE, RP-HPLC	LC-MS/MS and *de novo*	WAFAPA MYPGLA	661.3224 650.3098	Wang et al., 2018
Hairtail (*Trichiurus japonicas*) Muscle	Ultrafiltration membrane, AIEC, GFC, RP-HPLC	ESI-MS	AKG IYG	217.3 351.4 Da	Yang et al. (2019)

A = alanine, R = arginine, N = asparagine, D = aspartic acid, C = cysteine, E = glutamic acid, Q = glutamine, G = glycine, H = histidine, I = isoleucine, L = leucine, K = lysine, M = methionine, F = phenylalanine, P = proline, S = serine, T = threonine, W = tryptophan, Y = tyrosine, V = valine, GFC = Gel filtration chromatography, RP-HPLC = reverse phase-high performance liquid chromatography, FPLC = fast-performance liquid chromatography, UPLC = ultrahigh performance liquid chromatography, Q-TOF/MS = quadrupole time-of-flight, ESI = electrospray ionization, MS = mass spectrometer, LC-MS = liquid chromatography tandem mass spectrometer, MALDI-TOF-MS/MS = Matrix-assisted laser desorption/ionization-time of flight mass spectrometer.

##### 4.1.3.2 Identification of bioactive antioxidant peptides

Following consecutive purification processes, antioxidant peptides are usually taken to one of the different kinds of amino acid sequencers, such as liquid chromatography-tandem mass spectrometry (LC-MS/MS) (Mahdi et al., 2018) or ultrahigh performance liquid chromatography-tandem mass spectrometry (UPLC-MS/MS) (Zhang, Huang and Mu, 2019b). LC-MS/MS technique enables successful identification of antioxidant peptides using a quadrupole time-of-flight tandem mass spectrometer (Q-TOF) equipped with electrospray ionization (ESI) source, which runs in the positive ion mode (Chen et al., 2019). Alternatively, Matrix-assisted laser desorption/ionization-time of flight spectrometer (MALDI-TOF-MS/MS) can be used to identify antioxidant peptides, which have been purified from protein hydrolysates (Ketnawa et al., 2018).

### 4.2 Bioinformatic (*in silico*) approach

Bioinformatic is an innovative, supportive and systematic strategy developed to overcome cost-intensive and time-consuming conventional bioactive peptide investigation methods. This approach uses several databases, such as NCBI, BIOPEP-UWM and BLAST, containing different information about the amino acid sequence of a wide range of various parent proteins and peptides, frequency of bioactive peptides occurrence, and specific enzymes to be used for releasing peptides with predicted activities and properties. Furthermore, these databases can also be used to anticipate the physicochemical, functional and sensory properties of the predicted peptides.

The amino acid composition and sequence of the protein greatly determine the presence of antioxidant peptides with required bioactivities (Tejano et al., 2019). The amino acid sequence of the protein can be obtained from databases, such as UniProtKB and NCBI, if the protein source is studied and reported that the hydrolysate from this protein exhibited particular bioactivity. Darewicz, Borawska and Pliszka (2016) used UniProtKB database to retrieve the amino acid sequence of Carp protein for prediction of potential antioxidant peptides. Alternatively, proteomic techniques can be used to analyze and characterize amino acid sequences of unknown proteins from new source materials (Harvian and Ningrum, 2019). In recent years, a mass spectrometer (MS) based proteomics technique has been successfully used to determine and identify various proteins from food materials (Huang et al., 2015; Panjaitan et al., 2018; Tejano et al., 2019). The sequence obtained from MS techniques can be further observed using BLAST analysis to examine the homology of the identified protein by aligning with similar proteins of other materials obtained from NCBI database. BLAST analysis generates data of “identities”, “positives” and “gaps” of the two or more aligned proteins (Panjaitan et al., 2018).

Once the protein is identified and characterized, sequences are subjected to *in silico* analysis in BIOPEP-UWM database, where simulation of enzymatic hydrolysis is performed, to predict bioactive peptides theoretically released from the intact protein sequence, as well as potential enzymes possibly used to release the peptides (Darewicz et al., 2016). According to the study by Tejano et al. (2019), the application of BIOPEP-UWM database revealed that a high number of DPP IV inhibitors could be obtained from *Chlorella sorokiniana* proteins identified by LC-ESI-MS/MS. The study also used *in silico* analysis to simulate enzymatic hydrolysis and revealed that pepsin, bromelain and papain peptidases released a relatively large number of antioxidant peptides.

## 5 Emerging processing technologies for production of antioxidant peptides

Even though biochemical hydrolysis methods are the most commonly preferred strategies for the production of antioxidant peptides, the methods are still not feasible to produce bioactive peptides at the industrial level with higher yield and low cost. Accordingly, biochemical hydrolysis methods coupled with emerging processing technologies, such as high hydrostatic pressure (HHP), microwave, and pulsed electric field, have recently been considered to overcome the limitation associated with conventional methods (Dong et al., 2019; Gohi et al., 2019; Wen et al., 2019; Yang et al., 2017). As a result, these technologies have been found as the most promising methods to reduce the time and costs of biological agents used by biochemical methods alone, and improve the yield and bioactivities of peptides (Franck et al., 2018; Liu et al., 2019; Nguyen et al., 2017; Wang et al., 2017c).

### 5.1 High hydrostatic pressure processing

High hydrostatic pressure (HHP) is one of the novels non-thermal processing technologies in which isostatic pressure, typically 100–1000 MPa, is rapidly used and transferred to liquid or partially liquid-based products (Marciniak et al., 2018). In the past two decades, this technology has successfully been used in the food industry to improve the shelf life of food products with minimal effect on the nutritional values and sensory qualities of products. Moreover, the functional properties of food products can be improved by HHP because of structural modification in macromolecular components of the products (Franck et al., 2018). The protein conformations change and protein molecular chain extension, therefore, support the proteolysis reaction by allowing the enzymes to cleave peptide bonds in the new restriction sites and help to produce the bioactive peptides (Zhang and Mu, 2017). In this regard, HHP has attracted considerable interest in the area of proteomics.

Several recent studies testified that treatment of protein by HHP improves enzymatic hydrolysis, and antioxidant peptides yield and bioactivity (Bamdad et al., 2017a, Bamdad et al., 2017b; Dong et al., 2017; Garcia-Mora et al., 2016; Hu et al., 2017; Iskandar et al., 2015; Zhang and Mu, 2017; Zhang et al., 2016). According to these studies, the impact of high-pressure processing on the efficiency of enzymatic hydrolysis and bioactivity of antioxidant peptides depends on pressure level, holding time, and protein and enzyme type.

Bamdad et al. (2017b) reported that enzyme hydrolysis of casein protein under HHP at 100 MPa improved the degree of hydrolysis and antioxidant properties compared to 200 MPa and atmospheric pressure enzyme hydrolysis. Similarly, Bamdad et al. (2017a) revealed that HHP assisted enzyme hydrolysis of β-lactoglobulin protein at 100 MPa resulted in higher degree of hydrolysis, and enhanced free radical scavenging and reducing capacity than the hydrolysates produced with the same enzymes at atmospheric pressure. The most recent study by Dong et al. (2019) also demonstrated that the degree of hydrolysis, and reducing power and DPPH radical scavenging capacity of peanut protein hydrolysate treated at 300 MPa for 60 min and 100 MPa for 180 min are significantly higher than untreated and 500 MPa treated proteins. Based on the reports by these studies, high-pressure treatment of casein and β-lactoglobulin up to 100 MPa, and peanut proteins up to 300 MPa might change the protein conformation, which creates new cleavage sites for enzyme, and enhance enzymes activity and enzyme-substrate interactions (Zhang and Mu, 2017; Zhang et al., 2016). However, increasing the pressure levels for casein and β-lactoglobulin beyond 200 MPa and peanut protein 500 MPa could cause the formation of higher molecular weight proteins due to the re-associations of protein fragments (Dong et al., 2019; Garcia-Mora et al., 2016). The difference in the optimum pressure levels reported by Dong et al. (2019); Bamdad et al. (2017a) and Bamdad et al. (2017b) might be due to the differences in protein structures, protein-protein interactions and molecular weights of casein and β-lactoglobulin, and peanut protein (Garcia-Mora et al., 2016). Hydrophobic interactions, which play a major role in the stabilization of the tertiary structure, and protein-protein interactions, are the most pressure-sensitive followed by ionic and hydrogen bonds (Garcia-Mora et al., 2016).

Bamdad et al. (2017b) observed that a higher degree of hydrolysis and antioxidant capacity is achieved by HHP assisted flavourzyme treated casein hydrolysate than savinase, thermolysin and elastase enzyme-treated hydrolysates. Substantial differences observed between casein hydrolysates produced by flavourzyme and other enzymes for their degree of hydrolysis and antioxidant properties might be due to the differences in enzyme specificity and the magnitude of the catalytic activity increased as a consequence of pressure level (Garcia-Mora et al., 2016).

### 5.2 Microwave-assisted processing

In the last two decades, various biologically active compounds from a wide range of different plant, animal and marine resources have been successfully extracted using microwave-assisted processing technology (Gohi et al., 2019; Ketnawa and Liceaga, 2016; Nguyen et al., 2017; Zhang, Huang and Mu, 2019b). Microwave processing involves the use of electromagnetic radiation, in a frequency range of 300 MHz-300 GHz, to heat the solvent in the sample faster than the conventional heating method (Wang et al., 2017b). The mechanism of microwave-assisted extraction is through inter- and intra-molecular friction, together with the movement and collision of charged ions, causing rapid heating of the reaction system and resulting in the breakdown of cell walls and membranes (Jin et al., 2019).

As regards, several recent types of researches have been studied on the potential application of microwave-assisted processing in protein hydrolysate and the production of bioactive peptides. Accordingly, microwave heating has been found as a beneficial technique to enhance enzymatic proteolysis y reducing the hydrolysis time while improving the hydrolysate properties (Jin et al., 2019; Nguyen et al., 2015; Zhou et al., 2018). It is thought that pretreatment of protein by microwave heating changes the protein conformation and enhances the accessibility and susceptibility of the bonds to enzymes (Ketnawa and Liceaga, 2016; Zhang et al., 2019a, Zhang et al., 2019b).

According to the study by Ketnawa and Liceaga (2016), the application of microwave pretreatment, at 800 W and 90 °C for 5 min followed by conventional enzymatic hydrolysis with alcalase for 2–10 min, is found to increase the degree of hydrolysis, protein solubility and free radical scavenging activity of fish frame protein hydrolysate than the untreated hydrolysate. Similarly, Nguyen et al. (2015) reported that microwave intensified enzymatic deproteinization of Australian rock lobster shells significantly improved the degree of deproteinization from 58 to 85.8 % with excellent functional properties including protein solubility, oil absorption capacity and water absorption capacity. On the contrary, Zhang, Huang and Mu (2019a) demonstrated that steaming and autoclaving pretreatment significantly increased the degree of hydrolysis and antioxidant activities of sweet potato protein hydrolysate than microwave treated hydrolysates. Conventional heating methods can also enhance the degree of hydrolysis and antioxidant activity of protein hydrolysates because the unfolding of protein molecules increases the susceptibility of protein to enzyme hydrolysis (Zhang, Huang and Mu, 2019b). However, conventional hydrolysis methods need a longer time to obtain a higher degree of hydrolysis and antioxidant activity (Ketnawa and Liceaga, 2016).

### 5.3 Ultrasonic assisted processing

Like the other novel processing technologies, ultrasound processing has recently attracted considerable attention in enzymolysis to produce bioactive peptides (Dong et al., 2017; Wen et al., 2019; Wu et al., 2018; Zou et al., 2016, Zou et al., 2016). Two types of ultrasonic processing technologies, namely high intensity (16–100 kHz, power 10–1000 W/cm^2^) and low intensity (100 kHz–1 MHz, power <1 W/cm^2^) ultrasonic, are used in the food industry (Wang et al., 2017c). Acoustic cavitation of the ultrasound creates mechanical and thermal effects, which are the ultimate process responsible to modify the physicochemical properties of a material (Ozuna, Paniagua-martínez, Castaño-tostado, Ozimek, & Amaya-llano, 2015; Zou et al., 2015). Recently, several studies have provided evidence on the use of ultrasonic processing as a pretreatment operation to improve the protein hydrolysis procedure and increase the antioxidant activity of hydrolysates and peptides (Hayta and İşçimen, 2017; Ma et al., 2018; Sae-leaw & Benjakul, 2018; Wen et al., 2019).

Zou et al. (2015) revealed that ultrasound pretreatment of porcine cerebral protein at a single frequency of 20 kHz and a maximum power of 80 W for 5 min followed by alcalase digestion for 20 min is found to have higher peptides concentration (225.6 μg/mL) than the conventional alcalase hydrolyzed porcine cerebral protein (157.2 μg/mL) at the same hydrolysis time. Moreover, ultrasound pretreatment of porcine cerebral protein hydrolysate had higher reducing power activity and scavenging effect on DPPH radicals (72%), ABTS radicals (73%) and hydroxyl radicals (56%) at 2 mg/mL than the untreated samples. Similarly, Wang et al., 2017c reported that ultrasound treatment of β-conglycinin and glycinin proteins significantly increased the degree of hydrolysis, free SH groups, and iron chelating capacity of the protein hydrolysates. These could be because of the increase in the α-helix and β-turn proportions, and decrease in β-sheet and random coil proportions of the two protein fraction after ultrasonic treatment. Furthermore, ultrasound-assisted alcalase digestion enhances the antioxidant activity of corn protein hydrolysate because of the formation of short-chain peptides containing hydrophobic amino acids with a molecular weight of 200–3000Da (Liang et al., 2017, Liang et al., 2017).

### 5.4 Pulsed electric filed processing

Pulsed electric field (PEF) is non-thermal, low energy and a short time processing technology that has been widely used to sterilize, dehydrate and thaw foods, inactivate endogenous enzymes, mature wine, reduce food allergies and assist the extraction of bioactive compounds from different food-derived sources (Ghosh et al., 2019; Li et al., 2015; Polikovsky et al., 2016). PEF treatment is typically carried out at electric field strength of 10–50 kV/cm in multiple short pulses (typically 1–5 μs) at frequencies of 0.2–0.4 MHz. Cell disintegration through electroporation of the cell membrane is the key mechanism where PEF achieves the required functions in food products (Chian et al., 2019; Polikovsky et al., 2016).

In recent years, PEF processing has been reported to induce changes in the secondary and tertiary structure of proteins by making them lose their β-sheet and α-helix structures (Zhang et al., 2018). Pretreatment of proteins with PEF, therefore, exposes hydrolysis sites, which are previously inaccessible to digestive peptidases. Chian et al. (2019) observed that PEF treatment of bovine muscle caused disruption of Z disks and I bands (revealed by transmission electron micrographs) of the samples after 180 min of enzymatic digestion, thus, improved the susceptibility of the protein bonds to enzymatic degradation. Mikhaylin, Boussetta, Vorobiev and Bazinet (2017) also demonstrated that high voltage pulsed electric field treatment of β-lactoglobulin induced the formation of the active sites for the nucleophilic enzyme action. According to the most recent study by Liu et al. (2019), PEF treatment of ovomucin-depleted egg white analogously enhanced the antioxidant capacity of hydrolysate.

Although many studies investigated the effect of PEF as a pretreatment technique to improve the enzymatic hydrolysis procedure, PEF processing technology, more recently, has shifted its application from assisting the enzymatic hydrolysis procedure to improving the intracellular bioactivities of identified peptides. For example, the intracellular antioxidant activities of pine nut protein peptides Gln-Asp-His-Cys-His (Liang et al., 2017, Liang et al., 2017), Lys-Asp-His-Cys-His (Liang et al., 2019), Lys-Trp-Phe-Cys-Thr and Gln-Trp-Phe-Cys-Thr (Yang et al., 2017), and soybean protein peptide Ser-His-Glu-Cys-Asn (Lin et al., 2017) in HepG2 cells have been improved after treating the peptides by PEF processing technology.

It is well established that the activity of antioxidant peptides depends on several factors such as molecular weight, hydrophobicity, amino acid composition, and spatial conformation of the peptides. However, there are few reports on the possible mechanisms of how PEF treatment improves such antioxidant properties of the peptides. Zhang et al. (2018) investigated the possible mechanisms of how pulsed electric field treatment increases the antioxidant activity of Glu-Try-Phe-His peptide isolated from pine nut (*Pinus koraiensis*) protein. The study applied reversed-phase HPLC, Uv-vis spectroscopy, intrinsic fluorescence spectra, circular dichroism spectroscopy, and 1D and 2D NMR spectroscopies in series as exploration methods for the possible mechanisms. Finally, the study disclosed that the improvement of the antioxidant activity of Glu-Try-Phe-His peptide is because of the exposure of aromatic amino acids in the peptide solution, conversion of β-turn structure to random coil and unfolding of the peptides in the solution, and exposure of more active sites of the peptide to react with free radicals after the PEF treatment.

### 5.5 Subcritical water processing

Subcritical water processing is a green technology that employs the application of pressurized hot water generated by heating water at temperature levels between its atmospheric boiling point (100 °C, 0.1 MPa) and critical point (374 °C, 22.1 MPa) (Cho et al., 2018). At these subcritical points, surface tension and viscosity of the water decrease because of the change in its dielectric property, which makes it an excellent solvent to dissolve organic substances that are insoluble at the atmospheric boiling point of water (Park et al., 2019). Accordingly, supercritical water processing has been applied to produce antioxidant peptides from various protein sources (Ahmed and Chun, 2018; Álvarez et al., 2016).

The yield and bioactivity of subcritical water treated protein hydrolysate depend manly on hydrolysis temperature and pressure. Tuna skin protein and collagen hydrolysates treated by subcritical water at 280 °C and 80 bar showed the highest ABTS and DPPH radical scavenging activities, ferric reducing antioxidant power (FRAP) and metal chelating property (Ahmed and Chun, 2018). Ahmed and Chun (2018) also reported that the degree of hydrolysis of both samples increased with increasing temperature, with the highest degree of hydrolysis at 250 °C and 50 bar. The degree of hydrolysis of the two samples, however, decreased above a temperature of 250 °C because of the decomposition of amino acids due to excessive hydrolysis at very high temperatures. Álvarez et al. (2016) also reported that peptides obtained from subcritical water hydrolyzed porcine hemoglobin at 180 °C and 40 bar showed good antioxidant and functional properties with the highest yield (83%).

## 6 Conclusions and further perspectives

Nowadays, people all over the world are concerned about the rapid growth of non-communicable diseases, such as diabetes, cardiovascular diseases, blood pressure and cancers. Due to these facts, people's demand and interest for health-promoting foods have dramatically increased. Furthermore, technological advancement and demographic and socio-economic changes indicate the need for functional foods. Simultaneously, researchers have extensively conducted a wide range of different studies to find solutions for consumers' demand and interest in health-promoting foods. Isolation and production of antioxidant peptides, which are possibly used as ingredients for functional foods, have been identified as one of the potential ways to combat the occurrences of diseases because of reactive oxygen species (ROS).

Bioinformatic approach, for the isolation of antioxidant peptides, is a promising strategy designed to overcome cost-intensive and time-consuming conventional methods in a systematic process and method. However, bioactive peptides theoretically obtained using this approach may not be found the same when they are tested practically. This problem is not known briefly yet so that it needs further studies to understand the problem.

Emerging processing technologies coupling with the enzyme hydrolysis method has been found as a better method to produce antioxidant peptides in a short time and low cost. However, there are limited studies regarding the improvement of fermentation using novel processing technologies while fermentation is a promising method for the production of bioactive peptides at the industrial level. Therefore, fermentation of proteins treated with high pressure, microwave, ultrasound, pulsed electric field or subcritical water processing could be feasible to produce improved antioxidant peptides in a shorter time and lower cost than enzymatic hydrolysis of protein treated by novel processing methods.

Numerous studies have been conducted on the identification and evaluation of *in vitro* bioactivity of antioxidant peptides from protein hydrolysates of various protein sources. Most of these studies suggested that these novel ingredients could be used to produce functional foods. However, there are no or limited studies conducted on the application of antioxidant peptides in health-promoting foods development. Therefore, it is necessary to develop model functional foods containing antioxidant peptides and study the interaction of the peptides with other constituents of the model functional foods. Furthermore, the effects of incorporating these peptides and processing conditions on the bioactivity of the peptides in the food matrixes of model functional foods shall be investigated. Afterward, *in vivo* studies should be conducted to evaluate safety aspects, bioavailability and bioactivity of the peptides in the food matrixes.

## Declarations

### Author contribution statement

All authors listed have significantly contributed to the development and the writing of this article.

### Funding statement

This research did not receive any specific grant from funding agencies in the public, commercial, or not-for-profit sectors.

### Competing interest statement

The authors declare no conflict of interest.

### Additional information

No additional information is available for this paper.
